# Successful Management of Complications Due to a Cesarean Section in a Patient With a Complex Obstetric History: A Case Report and Review of Preventive Strategies

**DOI:** 10.7759/cureus.77183

**Published:** 2025-01-09

**Authors:** Esha Parikh, Samira Kanetkar, Reena Sheth, Renee Alexis

**Affiliations:** 1 Foundational Sciences, Nova Southeastern University Dr. Kiran C. Patel College of Osteopathic Medicine, Fort Lauderdale, USA; 2 Obstetrics and Gynecology, Nova Southeastern University Dr. Kiran C. Patel College of Osteopathic Medicine, Fort Lauderdale, USA

**Keywords:** abdominal hysterectomy, complicated cesarean, intra-abdominal adhesions, multigravida, severe dysmenorrhea, uterine fibroids in pregnancy

## Abstract

A multiparous 37-year-old female patient (G7P3A4) with a complex obstetric history consisting of a myomectomy for uterine fibroids and a previous cesarean section (C-section) resulted in complicated intra- and postpartum events. The incidental cystotomy during the C-section was managed conservatively by catheterization. Postoperatively the patient suffered from postpartum menorrhagia and an enlarged uterus and was managed with an explorative laparotomy concluding with extensive lysis of adhesions and total abdominal hysterectomy and bilateral salpingectomy. The patient did well postoperatively and was discharged after 48 hours. She returned for an outpatient follow-up one week later and was reassured that her pain would improve within six to eight weeks. This case study details the complications and aftermath of intense intra-abdominal adhesions and calls for a better understanding of the management of adhesions for abdominal procedures.

## Introduction

Cesarean section (C-section) is one of the most common abdominal surgical procedures performed on women. The rate of cesarean deliveries has increased globally, now accounting for more than 21% of all childbirths [1]. The procedure is associated with numerous postoperative complications, including infection, hemorrhage, and thrombosis. However, the most common complication of a C-section is abdominal adhesions or fibrous bands of scar tissue that form in response to tissue disruption during surgery. 

Adhesions typically form within the first few days after surgery but can persist for months or even years following a procedure. Most abdominal adhesions are asymptomatic. When symptoms do occur, they can present clinically as chronic abdominal or pelvic pain, nausea, vomiting, abdominal distension, dyspareunia, or changes in bowel movements [2].

Studies have shown that in women who had a history of a C-section, adhesions could be seen in 24% to 65% [3]. The most important predictor of adhesions is a history of cesarean deliveries. One study found an increased rate of abdominal adhesions among patients with four or more prior C-sections than those who had undergone three or fewer [4]. Additionally, estimates of adhesions following the first C-section ranged from 24.4% up to 73% when the parietal peritoneum was left open, suggesting that the method of wound closure could affect the severity of adhesions [5].

Diagnosis is often accidental, as most cases of abdominal adhesions are asymptomatic. Laparoscopy is the diagnostic gold standard, but less invasive methods like transvaginal ultrasound can be used to visualize the free movement of the abdominal wall [6]. Treatment involves surgery to release adhesions and remove possible obstruction. Since the laparoscopic technique is minimally invasive, it is preferred as it allows easy access to the abdominal cavity and enables visualization of the adhesions. In some cases where adhesions are severe or inaccessible, open surgery may be necessary.

## Case presentation

Our case focuses on a 37-year-old African American female patient with a history of seven total pregnancies, three of which were full-term live births and four spontaneous abortions (G7P3A4). Additionally, there was a medical history of fibroids and a surgical history of two C-sections and one myomectomy, which triggered severe adhesion formation to the lower abdominal wall of the uterus. 

**Figure 1:**
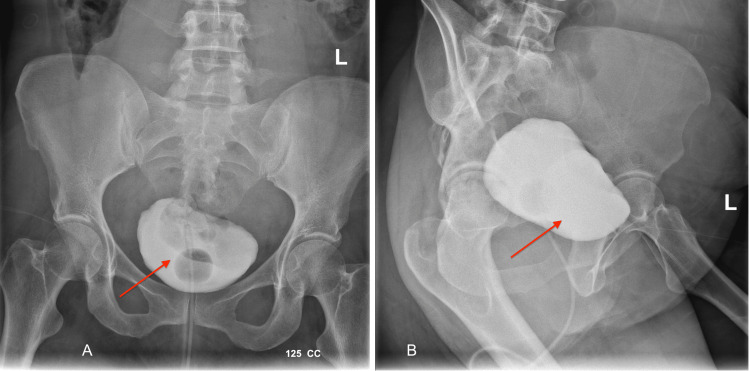
Retrograde cystogram five weeks after the cesarean section The red arrow shows an intact bladder.

The patient's surgical history confirmed the possibility of adhesion between the uterus and the urinary bladder. The situation is further complicated with the incidental cystotomy requiring the placement of an indwelling catheter. Later, the retrograde cystogram confirmed the healed bladder (as seen in Figure 1). However, lower abdominal pain and uterine bleeding persisted.

Computed tomography scans done 4.5 weeks after the C-section showed an enlarged uterus with fibroids and free fluid with gas in the extraperitoneal cavity between the anterior abdominal wall and uterus, suggesting dehiscence of the C-section incisions. The patient was managed conservatively with plans to follow up for a repeat CT scan in a month. 

**Figure 2:**
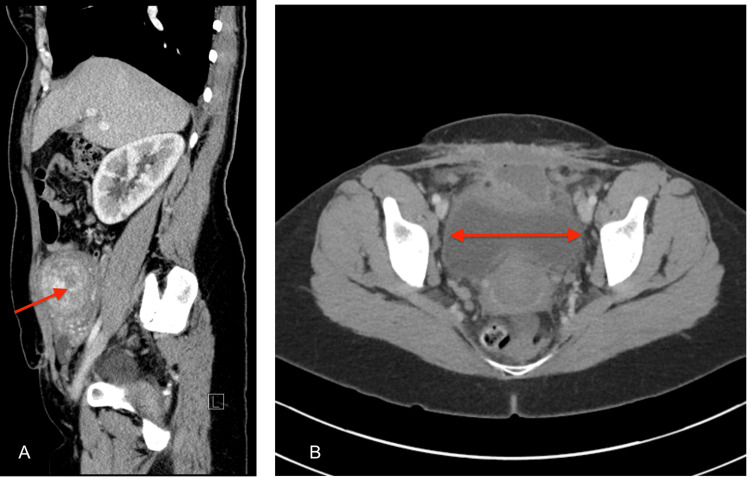
A CT scan of the abdomen and pelvis eight weeks before total abdominal hysterectomy The red arrow shows the uterine fibroid.

On the CT scan eight weeks after the C-section, the uterus was enlarged, measuring 12.5 x 9.1 x 13.5 cm, and contained multiple myometrial fibroids; the largest one was 2.7 cm, as seen in Figure 2. Imaging also showed fluid present in the extraperitoneal area with no additional peritoneal thickening anterior to the uterus and urinary bladder measuring 7.5 x 1.0 cm. Mild bilateral hydronephrosis and 10 mm cortical cysts in the right kidney were observed, possibly due to extrinsic compression from the enlarged uterus. 

The patient continued to have symptoms of dysmenorrhea and developed anemia. On a physical exam, the uterus was palpable through the umbilicus, consistent with a uterine size of 20 weeks gestation. On account of the patient's grave symptoms, a total abdominal hysterectomy and bilateral salpingectomy were decided. 

A surgical plan was coordinated with the general surgeon to assist with an exploratory laparotomy with extensive lysis of adhesions. The anterior preperitoneal dense adhesions were initially lysed, separating the bladder and uterus, which was followed by an incidental appendectomy. Following this, the adhesions were dissected up the sigmoid colon for a length of 4-5 inches. This was continued posteriorly, revealing small bowel and rectal adhesions, which were lysed to create the rectouterine pouch. With the space visible, the uterus was completely dissected off the anterior bladder. Both ovaries were preserved, given the patient's age. 

The surgery was completed without complications and addressed the symptoms of dysmenorrhea and anemia. Additionally, the lysis of adhesions and incidental appendectomy decreased the risk of potential risks of small bowel obstruction or another abdominal surgery.

## Discussion

Intra-abdominal adhesions are common and often debilitating complications of uterine surgeries, specifically repeat C-sections, with an increased likelihood of bladder injuries following procedures, as demonstrated in this patient [7]. Studies have shown that approximately 74.32% of patients undergoing repeat C-sections developed intra-abdominal adhesions, which are attributed to abnormal healing, where fibrin clots are not fully degraded, forming a fibrous network [8,9]. 

The burden of adhesions extends beyond physical and mental obstacles; patients and the healthcare systems face considerable financial stress. Data have shown that patients with intra-abdominal adhesions have prolonged hospital stays, longer recovery times, and warranted delivery from expert surgeons [10]. The UK Surgical and Clinical Adhesions Research (SCAR) group revealed that 5.7% of all readmissions after open abdominal or pelvic surgeries were due to adhesions. These adhesions are responsible for various complications, including 15%-20% of female infertility cases, 80% of chronic postoperative abdominal pain, and 60% of obstructions, significantly affecting the technical difficulty of subsequent surgeries. Adhesiolysis procedures alone cost the U.S. healthcare system over $2.3 billion in 2005, with the total economic burden of adhesion-related issues, excluding outpatient care and productivity losses, estimated to be much higher, including a direct hospital cost of $3.45 billion for adhesive small bowel obstruction in the same year [6]. This was evident in our patient, who required a cystogram following her last C-section, subsequently leading to a supra-abdominal hysterectomy, all within a mere eight-week period. 

Recognizing this burden makes it essential to explore preventative and diagnostic practices for intra-abdominal adhesions. Recent advances have shown that bipolar energy systems can decrease intra-uterine adhesions (IUA) by up to 7.5% and cold loops by up to 4.2%. Employing barrier methods postoperatively aimed at separating the uterine wall from the small bowel and pelvic organs would have been a viable option for this patient after her initial myomectomy. Gels, particularly hyaluronic acid, have been shown to reduce IUA and severity significantly [11]. Furthermore, the use of the 4DryField® PH adhesion barrier in patients undergoing surgery for endometriosis has shown a significant reduction in adhesion formation of 85% when compared to a control group that received only saline solution [12]. In patients undergoing surgeries like myomectomy, the use of oxidized regenerated cellulose has led to a 15.9% reduction in adhesions [13]. These highlight the potential for adhesion prevention strategies, which could be applied to C-sections and other gynecological surgeries to reduce the incidence and severity of intra-abdominal adhesions.

When preventative measures are insufficient, early diagnosis of intra-abdominal adhesions can help with proactive management. Cystoinflation has been shown to be beneficial in patients with a history of large fibroids and previous surgeries; the inflation of the bladder allows better visualization through the dense adhesions. In a randomized controlled trial, a highly significant decrease in bladder injury rates was observed between the control and cystoinflation groups. Specifically, the bladder injury rate was 20.6% in the control group compared to just 2.8% in the cystoinflation group. Additionally, bladder injuries in the cystoinflation group were significantly smaller in size, demonstrating that cystoinflation not only reduces the likelihood of bladder injury but also leads to less severe injuries when they do occur [14]. While this technique is promising, its standardization, training requirements, and feasibility in emergent surgeries remain challenging. 

A prospective study with 164 patients showed “sliding sign” as a cost-effective method. The process involves using an ultrasound over a transverse skin scar, followed by the patient taking a deep breath, and the uterus sliding under the peritoneum is observed [15]. This process was further supported by a retrospective study showing 16 of 19 patients labeled high risk due to absent sliding scales indeed had severe adhesions [16]. In addition, preoperative assessment of striae severity using the Davey score and C-section scar severity using the Vancouver Scar Scale have shown highly significant predictive performance [17]. These simple and non-invasive tools would help preoperative planning and avoid adhesion-related complications.

This case emphasized the need for standardized guidelines to prevent and manage intra-abdominal adhesions, specifically in patients with a history of multiple C-sections and other gynecological surgeries. Implementing preventative measures, improving diagnostic techniques, and optimizing surgical practices can help better patient outcomes.

## Conclusions

Intra-abdominal adhesions remain a common postoperative complication for patients who undergo obstetrical and gynecological procedures, such as cesarean section or myomectomy. For repeat C-sections, obstetricians should consider alternative surgical advances like bipolar energy systems and barrier methods that can significantly reduce intra-abdominal and IUA and their severity. Additionally, because diagnosis is made primarily by history and clinical presentation, obstetricians should exercise a high index of suspicion with patients who have a birth history of multiple cesarean deliveries. Improved guidelines for early recognition of adhesions are needed to prevent postoperative symptoms and reduce loss of reproductive years. It would also be beneficial to explore innovative surgical approaches that can reduce intra-abdominal adhesions, improving postpartum outcomes for mothers who delivered by C-section.
